# Transcatheter aortic valve-in-surgical aortic valve for a patient with repeated healed endocarditis: a case report

**DOI:** 10.1186/s40792-023-01739-z

**Published:** 2023-09-04

**Authors:** Yusuke Takei, Ryujiro Suzuki, Ikuko Shibasaki, Michiaki Tokura, Takahisa Nasuno, Hiroko Yazawa, Mayo Wada, Fumiya Saito, Shigeru Toyoda, Hirotugu Fukuda

**Affiliations:** 1 Department of Cardiac and Vascular Surgery, Dokkyo Medical University Graduate School of Medicine, Mibu-Machi, Shimotsugagun, Tochigi Japan; 2 Department of Cardiovascular Medicine, Dokkyo Medical University Graduate School of Medicine, Mibu-Machi, Shimotsugagun, Tochigi Japan

**Keywords:** Transcatheter valve replacement, Transcatheter aortic valve in a surgical aortic valve, Stentless valve, Prosthetic valve endocarditis, Healed infective endocarditis

## Abstract

**Background:**

Transcatheter valve replacement is contraindicated in patients with active infective endocarditis. However, few reports suggest that it could be beneficial for high-risk surgical patients with healed infective endocarditis. Here, we report a case of a surgical transcatheter aortic valve in a patient with healed repeated prosthetic valve endocarditis using a stentless valve.

**Case presentation:**

A 79-year-old female who underwent the Bentall procedure using a stentless valve and coronary artery bypass grafting for annuloaortic ectasia 22 years ago was hospitalized for stage II bioprosthetic valve failure. The patient had a history of prosthetic valve endocarditis three times: the first and second prosthetic valve endocarditis occurred 15 years ago, and the third prosthetic valve endocarditis occurred 3 years ago. The causative organisms were *Campylobacter fetus* and *Enterococcus faecalis*. With appropriate antibiotic therapy, the lesion was localized and healed completely without valve destruction; however, the patient developed rapid aortic regurgitation. Based on a review of the patient’s history of prosthetic valve endocarditis, the absence of signs of infection, and clinical findings of transesophageal echocardiography and computed tomography, a diagnosis of structural valve deterioration with healed infective endocarditis was made. Subsequently, a transcatheter aortic valve in a surgical aortic valve using a balloon-expandable type was performed, because the patient had a high surgical risk of 12.7%. The patient’s postoperative course was uneventful. At the 1-year follow-up, there were no signs of infection or valve abnormalities.

**Conclusions:**

Transcatheter valve replacement can be a treatment option for high-risk surgical patients with healed limited lesions in infective endocarditis.

**Supplementary Information:**

The online version contains supplementary material available at 10.1186/s40792-023-01739-z.

## Background

Infective endocarditis (IE) practice guidelines in the United States, Europe, and Japan recommend a combination of surgery and antibiotics as the gold standard treatment for IE, not only in urgent cases, such as heart failure, refractory disease, heart valve destruction, and embolism, but also when the heart valve remains destroyed after antibiotic treatment, especially in prosthetic valve endocarditis (PVE) [1–3]. Transcatheter valve replacement (TAVR) without the removal of infected valve leaflets and abscesses is generally contraindicated because of the risk of infection recurrence and the spread of vegetation throughout the body by manipulation. However, there are limited reports of TAVR as a bridge therapy or salvage [4, 5] for patients who are in very poor general condition and have low tolerance for surgery, especially in active aortic valve (AV)-IE, even though TAVR is now widely used. Apart from active AV-IE, TAVR for “healed IE” is controversial and may be effective in a limited number of cases [6]. In this report, we describe a case of transcatheter aortic valve implantation in a surgical aortic valve (TAV-in-SAV) in a patient with repeated PVE of a stentless valve.

## Case presentation

**Fig. 1:**
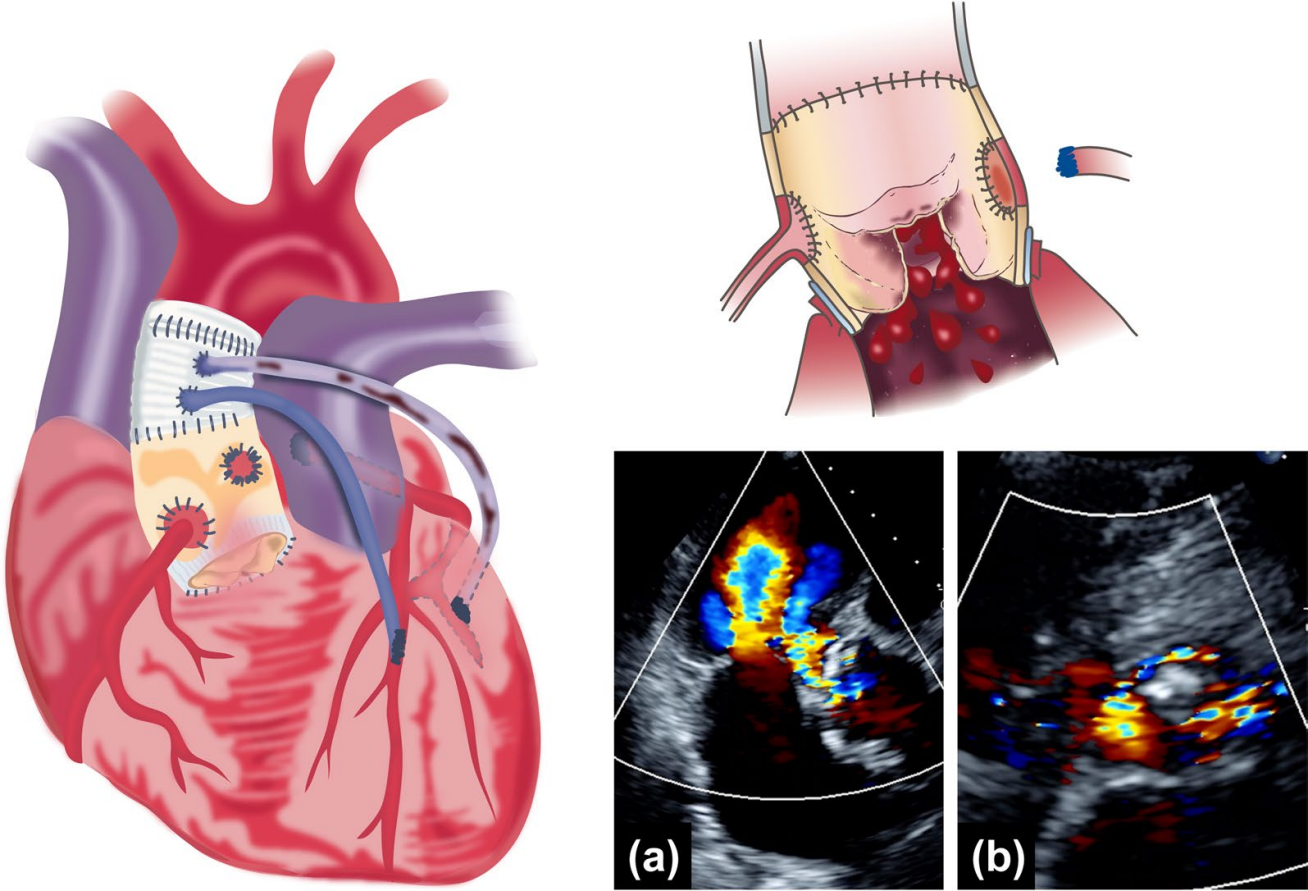
Previous heart surgery schema and TTE findings. The Bentall procedure was performed with a stentless valve, the left main trunk was sutured and ligated, and CABG was added to the LAD and OM with an SVG, because the left coronary bottom was too fragile to be reconstructed. The graft for the OM was later occluded by CT. The TTE findings show (**a**) severe AI and (**b**) hyperechoic lesions in the left cusp. The upper-right illustration was created based on the central illustration in the paper reported by Miller et al. [10]. *CABG* coronary artery bypass grafting, *LAD* left anterior descending artery, *OM* obtuse marginal branch, *SVG* saphenous vein graft, *CT* computed tomography, *TTE* transthoracic echocardiography, *AI* aortic insufficiency

A 79-year-old female, who had undergone Bentall with a stentless valve (23 mm Freestyle Aortic Root Bioprosthesis, Medtronic, Dublin, Ireland) in a full-root fashion and accidental coronary artery bypass grafting for annuloaortic ectasia 22 years earlier, presented with dyspnea upon exertion and orthopnea at night. Subsequently, she was referred to our hospital for further evaluation and treatment of heart failure based on a highly inactive N-terminal fragment of pro B-type natriuretic peptide (BNP) level of 2614 pg/mL (normal < 450 pg for adults 75 years or older) and mild pulmonary congestion found on chest radiography. On the transthoracic echocardiography (TTE), the family physician noted a severe aortic insufficiency (AI) associated with an adhesion of structures to the valve leaflets, which appeared to be vegetation and destruction of the prosthetic valve leaflets (Fig. 1). The patient had bronchial asthma under medical control and had undergone several surgeries other than cardiac surgery, such as ablation for arterial fibrillation, cholecystectomy, appendectomy, total hysterectomy for uterine cancer, and endoscopic submucosal dissection for early stage gastric cancer. She had been hospitalized for PVE three times in approximately 15 years. During an outpatient visit 1 month prior to admission, the patient did not report any shortness of breath or fever. The BNP level was 36.1 pg/mL (normal, < 100 pg/mL), and routine TTE revealed no vegetation or signs of AI.

**Fig. 2:**
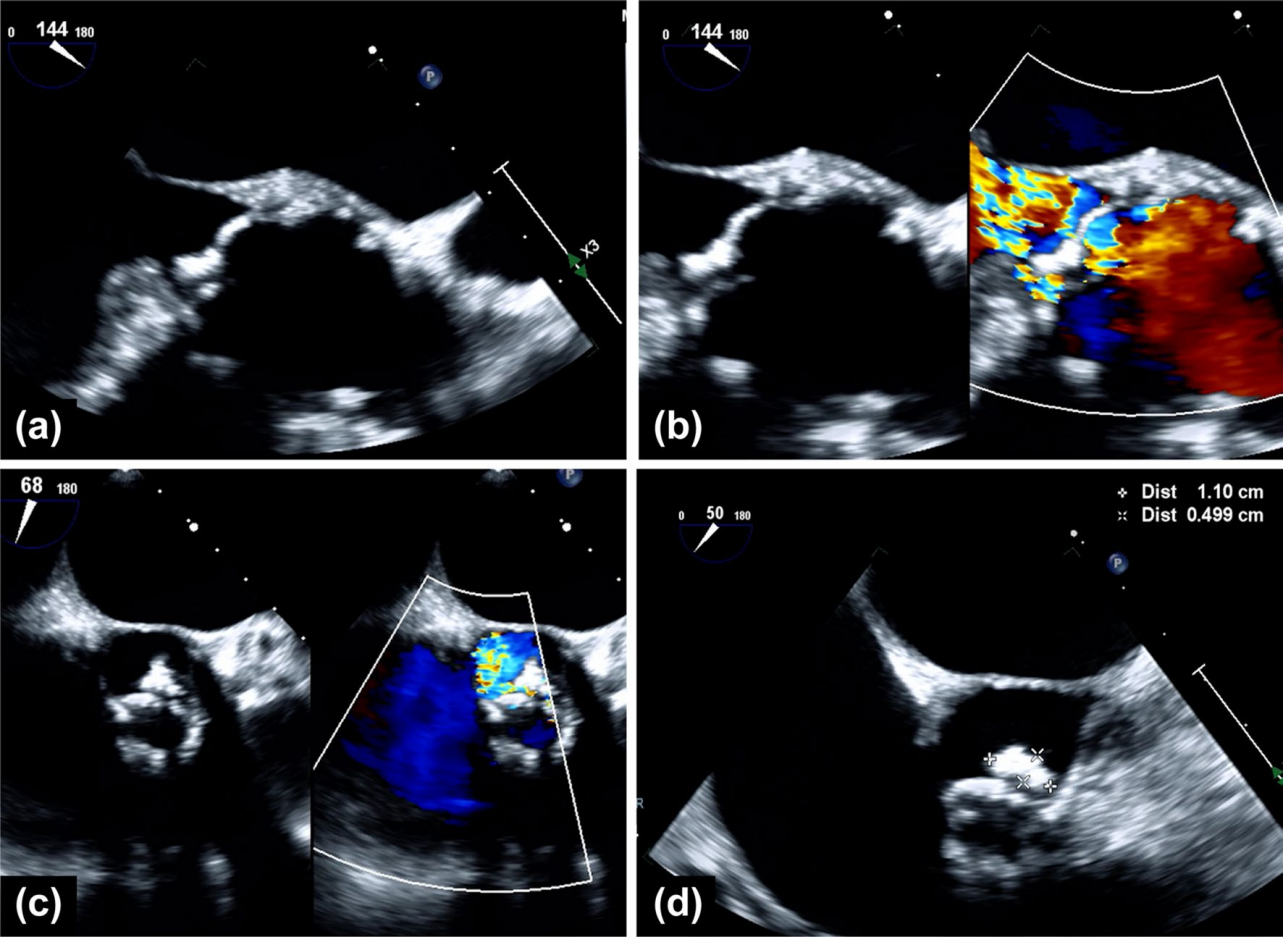
TEE findings. TEE showing prolapse of the thickened left cusp and severe AI in **a**–**c**. In addition, an oval structure of approximately 11 mm × 5 mm was attached to the left cusp (**d**). *TEE* transesophageal echocardiology, *AI* aortic insufficiency

Upon admission, the patient appeared underweight (height: 147.5 cm, weight: 36.6 kg, body mass index: 16.8 kg/m^2^) and exhibited general fatigue, but was afebrile. During physical examination, a loud, grade 4/6 diastolic murmur in the second intercostal space without crackles was identified on auscultation. Initial laboratory results revealed a hemoglobin level of 12.3 g/dL, white blood cell count of 3,300 /µL, a slightly elevated C-reactive protein of 0.47 mg/dL (normal, < 0.30 mg/dL), a prolonged prothrombin time-international normalized ratio of 3.1 for warfarin taken for atrial fibrillation and an elevated BNP level of 501 pg/mL. The blood culture results were negative. Chest radiography revealed mild bilateral pulmonary edema with cardiomegaly (CTR 58.9%). TTE revealed severe AI with left cusp prolapse and a reduced ejection fraction of 45%. Transesophageal echocardiography (TEE) revealed prolapse of the thickened left cusp with an oval structure of approximately 11 × 5 mm (Fig. 2 and Additional file 1: Video 1). No abnormalities were observed in the mitral or tricuspid valves. Evidence of infection was absent; however, the oval shape could not be definitively excluded as vegetation, given the patient's history of three instances of PVE. Therefore, the patient was tentatively diagnosed with stage III bioprosthetic valve dysfunction with severe valve morphology deterioration due to structural valve deterioration (SVD) with healed IE, according to the Valve Academic Research Consortium-3 [7]. The patient initially refused surgical treatment despite presenting with stage II bioprosthetic valve failure, but consented to surgical treatment after medical therapy. After initial treatment with additional diuretics (furosemide intravenous injection and tolvaptan were prescribed), contrast-enhanced computed tomography (CT) revealed that the right coronary artery (RCA) and saphenous vein graft to left anterior descending artery (SVG–LAD) bypass graft were patent, but saphenous vein graft to obtuse marginal artery bypass graft was occluded, indicating that the left coronary artery was perfused only by SVG–LAD bypass. The oval structure observed in the TEE could not be identified. Regarding the measurement of the stentless valve, the inflow diameter was 20.7 × 23.8 mm (area: 383 mm^2^), the outflow diameter was 27.1 × 29.6 mm (area: 648 mm^2^) and the sinus of Valsalva was 37.3 mm in left coronary sinus, 38.0 mm in right coronary sinus, 35.9 mm in non-coronary sinus, respectively, without pseudoaneurysm. The right coronary orifice height was 24.1 mm, and the proximal anastomosis of the SVG–LAD bypass height was 40 mm from the basal plane. The patient’s calculated operative risk was high according to the Society of Thoracic Surgeons Predicted Risk of Mortality (12.7%), and the clinical frailty scale was grade 3 [8]. In the initial discussion with the heart team, some participants deemed TAV-in-SAV suitable for SVD with healed IE, whereas others advocated revision despite the high surgical risk, citing concerns about PVE and recurrent infections. Hence, the potential of TAV-in-SAV was discussed again after investigating the patients’ PVE episodes.

### Investigation

**Fig. 3:**
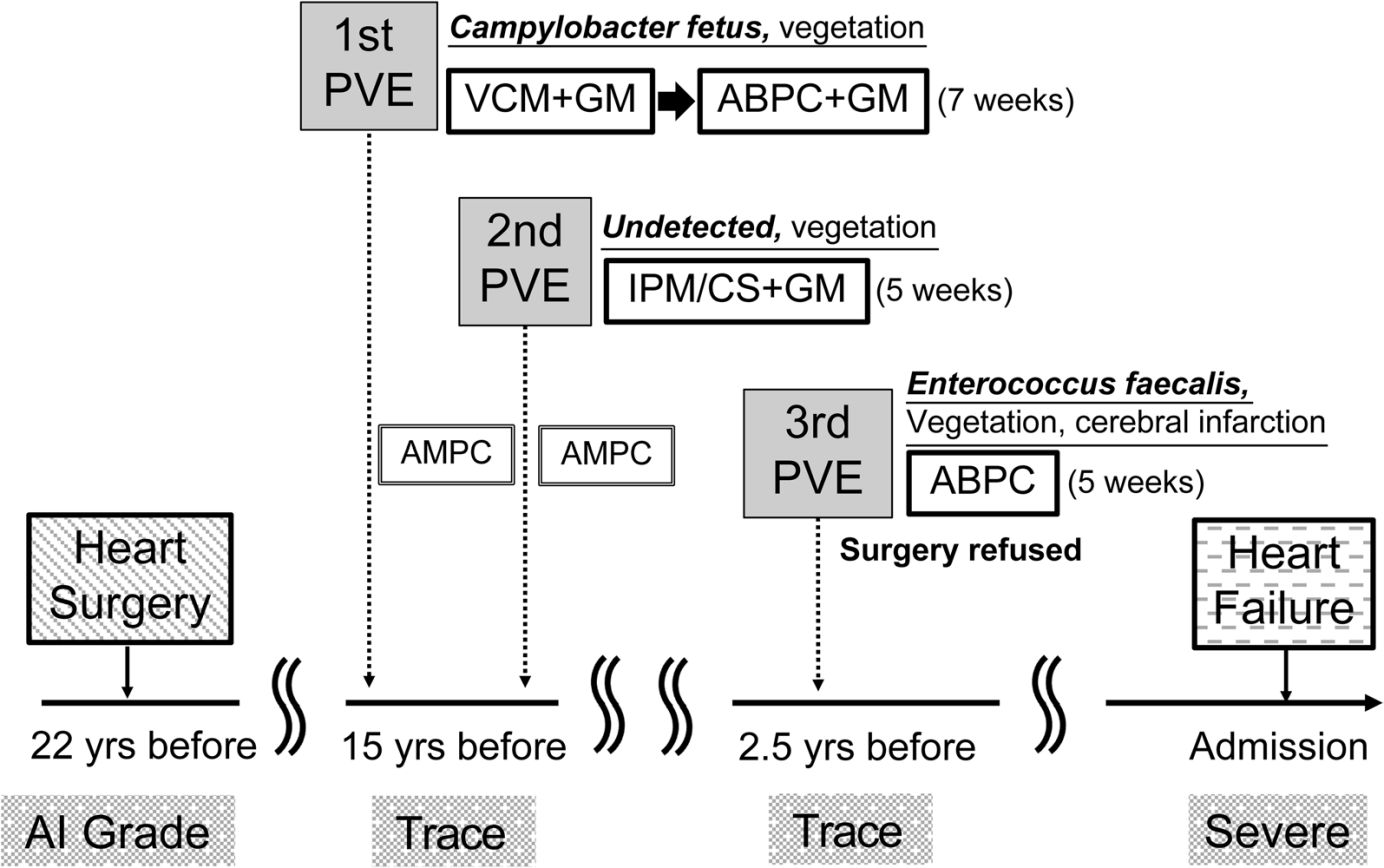
Treatment timeline for PVE. *PVE* prosthetic valve endocarditis, *VCM* vancomycin hydrochloride, *GM* gentamicin sulfate, *ABPC* ampicillin sodium, *IPM/CS* imipenem/cilastatin, *AMPC* amoxicillin hydrate, *AI* aortic insufficiency

The patient timeline for previous PVE treatment and TEE inspection is shown in Fig. 3 and Additional file 2: Video 2. Seven years after the cardiac surgery, the patient experienced an initial episode of PVE with fever, toothache, and sore throat. TTE and TEE revealed vegetation in the left cusp leaflet of the stentless valve, and *Campylobacter fetus* was detected in two sets of blood cultures, leading to a diagnosis of PVE according to the modified Duke’s diagnostic criteria [9]. Vancomycin hydrochloride and gentamicin sulfate were initiated as empirical antibiotics, transitioning to ampicillin sodium and gentamicin, respectively, once the causative organism was identified. The symptoms quickly subsided after antibiotic administration, with trivial AI, prompt disappearance of the vegetation, and no evidence of embolization. The patient was cured after 7 weeks of antibiotic therapy and discharged on an oral amoxicillin hydrate regimen. The second PVE diagnosis occurred 2 months after discharge (1 month after amoxicillin hydrate discontinuation), again presenting with fever and slight vegetation in the left leaflet valve. Blood cultures drawn during the hospital stay were negative. Although the causative organism could not be identified, the patient was treated for 5 weeks with imipenem/cilastatin sodium and gentamicin, which treats *Campylobacter fetus*, the causative organism of the previous PVE, and has a broad spectrum. Following treatment with amoxicillin, the vegetation disappeared, which was considered completely cured, and the patient was discharged from the hospital. There was no recurrence of the PVE over the next 11 years; however, fever and vegetation were detected on the right cusp. *Enterococcus faecalis* was identified in the blood culture and a third PVE was diagnosed. The patient had an acute cerebral infarction viewed on magnetic resonance imaging and was determined to have a class IIa indication for early revision surgery. The patient refused surgery and was discharged after 5 weeks of antibiotic therapy (ampicillin sodium); however, the vegetation disappeared, the blood culture was negative, the infection resolved, and there was no evidence of AI. For two and a half years, there was no recurrence of PVE, and no AI was observed in the annual TTE. The patient subsequently developed AI with sudden symptoms of heart failure.

Upon thorough examination of the patient’s history of PVE and considering the TEE and CT findings, we diagnosed the patient with healed IE. Given the potential benefits, we opted for TAVI-in-SAV over revision surgery despite little concern about PVE relapse in light of the patient’s present condition and preferences.

### Treatment (Fig. 4 and Additional file 3: video 3)

**Fig. 4:**
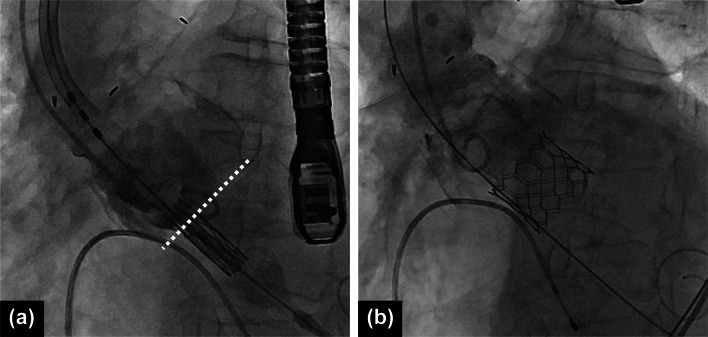
Transcatheter aortic valve in the full root stentless valve. The dotted lines show the nadir of the stentless valve leaflets. The center marker of Sapien3 was placed 1 center marker below the nadir (**a**) The final angiogram (**b**) after post-dilatation shows Sapien3 anchored to the sewing cuff of the stentless valve and a trivial paravalvular leak

We selected a balloon-expandable transcatheter heart valve (THV) (Sapien3, Edwards Lifesciences, Irvine, CA, USA) because of its implantability as a stentless valve [10]. TAV-in-SAV was performed using the right transfemoral approach under general anesthesia with TEE insertion. A pigtail catheter for angiography was inserted through the left femoral artery and positioned in the right coronary cusp. Subsequently, angiography was used to confirm the nadir of the stentless valve leaflets. During rapid pacing, a 23-mm Sapien3 valve was deployed. The center marker of the valve was shifted from the nadir line by one marker towards the left ventricular side to anchor it to the Dacron sewing cuff. TEE immediately after deployment showed a mild paravalvular leak (PVL); therefore, a post-dilatation balloon was inflated to the same pressure. The PVL was reduced to a trace, and the operation was completed within 1 h without complications, such as stroke, vascular injury, or conduction disturbance.

### Outcome and follow-up

The patient’s postoperative course was uneventful, and the patient was discharged 12 days after TAVR after gaining the same activity level as before surgery through rehabilitation. No antibiotics were administered during hospitalization, except 3 g/day cefazolin sodium, which was administered immediately before and 2 days after surgery as standard antibiotic prophylaxis. Six months postoperatively, the patient suffered a cerebral hemorrhage without disability caused by a prolonged prothrombin time-international normalized ratio. Despite requiring rehospitalization, the patient is currently doing well. At the 1-year postoperative outpatient follow-up, there were no chief complaints or signs of infection, the BNP level decreased to 105 pg/mL, and TTE showed no elevated pressure gradient or abnormal change in the valve (ejection fraction, 55%; mean transvalvular gradient, 9 mmHg; effective orifice area, 1.97 cm^2^; PVL, trace).

## Discussion

The discussion point in this case was whether TAV-in-SAV for SVD with healed PVE was the optimal option for the patient despite concerns about the potential relapse of PVE. Santos-Martinez et al. [6] reviewed TAVR for healed IE and determined that healed IE was defined as three consecutive negative blood cultures, no clinical or laboratory signs of sepsis, and no cardiac vegetation or abscess; this study included 54 patients, 26 of whom had a PVE. The treatment duration from the initial IE diagnosis to TAVR was 90 days (range: 21–411 days). *Enterococcus* spp. are the most commonly identified causative pathogens. Postoperatively, a high number of patients (18.5%, *n* = 10) developed sepsis, primarily due to respiratory infections. Among these cases, only one (1.8%) showed definitive recurrence of IE caused by methicillin-resistant *Staphylococcus aureus* (MRSA). The authors concluded that TAVR could be a valid therapeutic choice for selected IE patients with a low risk of local infection and high surgical risk. Determining whether the lesion is localized is important, and it may be useful to check for mitral valve involvement using TEE or evaluate it qualitatively using positron emission tomography–CT [6, 11]. Therefore, MRSA infections should be avoided. In this case, the patient experienced three episodes of late PVE. The first PVE was attributed to *Campylobacter fetus* and the third to *Enterococcus faecalis*, while the second had a negative blood culture result. During each PVE treatment course, the diagnosis was made promptly after onset, a thorough TEE evaluation was performed, and appropriate antibiotic therapy was administered to successfully localize the infection. However, the valve was found to have severe AI due to age-related deterioration, along with mild damage resulting from previous occurrences of PVEs. There was no evidence of mitral valve infection spillover during the course of PVEs. The use of a stentless valve without a surgical cuff may have contributed to localization of the infection. Based on this suggestion, the patient's preoperative findings fit the definition of healed IE. However, a preoperative positron emission tomography–CT scan should be performed to eliminate the potential risk of PVE. TAV-in-SAV was completed without any perioperative complications 1 year after surgery, and there was no recurrence of infection during the strict postoperative period.

Experienced high-volume centers have reported a 30-day mortality rate of 2–5% in young patients with a low operative risk for the re-do Bentall procedure [12–14]. Upon reconsidering the option of revision for this patient with high surgical risk, two factors arose that led to hesitation. The first cause of concern was the complexity of the patient's prior surgery, which involved suture ligation of the left main trunk and CABG, raising concerns regarding severe adhesions and potential graft damage. The second cause of concern was the patient being underweight, which has been reported to increase the re-exploration rate and mortality [15, 16]. Revision was indicated for this patient, not only at this time, but also at the third PVE because of cerebral infarction by vegetation. A revision procedure for PVE has a high operative mortality rate of 20–29% [17], which can be attributed to the patient's preoperative background, especially advanced age, comorbidities, MRSA infection, persistent sepsis, and extensive surgery for valvular abscesses [18]. However, approximately half of these patients choose conservative treatment because of serious complications (such as intracranial hemorrhage and severe sepsis) or refusal due to the perceived surgical risk. In the present case, the patient refused surgery due to the high risk of surgery, but fortunately, the lesion was localized and successfully treated with antibiotics alone. Brankovic et al. reviewed [5] six cases of TAVR for active IE (one native IE and five PVEs). These procedures were performed as rescue therapies for cardiogenic shock caused by heart failure with a high surgical risk. No deaths, strokes, or cardiac-related complications occurred within 30 days of the procedure, although some patients had vegetation. While the general use of TAVR for active IE is not widely supported due to the limited number of cases, short observation period (6–18 months), and risk of recurrent infection, it can be considered an option to improve heart failure and act as a bridge to re-do surgery for a limited number of patients.

The stentless valve lacks a frame for THV fixation and radiopaque markers for precise positioning, making TAV-in-SAV for stentless valves more complex than one for stented valves [19]. In addition, the main cause of failed stentless valves is AI without calcification, which further complicates implantation [20]. For the Freestyle valve, targeting the Dacron cuff below the leaflet attachment is crucial. In the current case, we confirmed nadirs using the pigtail catheter placed in the right coronary cusp. If challenging, additional pigtails or wires would confirm coplanar view [21]. With the precise location of the Dacron cuff eluding fluoroscopy confirmation, we resorted to measurements from the preoperative CT scan to determine the distance to the nadir and finalize the implantation site. TAV-in-SAV for a Freestyle valve, especially in a subcoronary manner, risks coronary obstruction [10]; however, in this case, full root manner and left coronary bypass lowered risk. Although self-expandable THVs that can be repositioned are generally preferred for stentless valves [21], we chose a balloon expandable THV, because self-expandable THVs are prone to malposition [20].

We had concerns about potential SVD of the implanted THV, considering the patient’s life expectancy. Another primary concern was the possibility of recurrent IE, given the patient’s history of three PVE episodes, although no recurrence occurred within 1 year after TAV-in-SAV. To address potential SVD, the option of repeat TAVI utilizing a self-expandable THV could be considered owing to the initial use of a 23-mm Sapien3 valve. The risk of coronary obstruction is minimal because of the adequate height between the proximal anastomosis of the RCA and SVG–LAD bypass and the valve implantation site. In addition, given the patient's small body size, the likelihood of a patient-prosthesis mismatch is low. In cases of uncontrolled IE recurrence, simultaneous redo-Bentall and CABG would be challenging, although both are invasive and risky procedures for this patient. However, conservative treatment may be necessary, depending on the patient's age and tolerance. According to the study from the Swiss TAVI registry, the overall incidence rate of PVE in patients undergoing TAVR over a 5-year follow-up period was reported as 1.0 events per 100 person-years [22]. Specifically, within 100-day post-implantation, the highest incidence rate has been reported as 2.59 events per 100 person-years. In addition, patients with TAVR-associated PVE have an approximately sevenfold increased risk of mortality and fourfold increased risk of stroke [22]. Surgical exploration of the THVs, which includes half of the indications for IE, is associated with poor outcomes, with reported mortality rates of 11.9% in hospitals, 13.1% at 30 days, and 28.5% at 1 year [23]. Notably, implantation of self-expandable THVs may necessitate intervention in the aortic structure and ascending aorta [24]. Considering these factors, we selected balloon-expandable THVs. We anticipated that, if a self-expandable THV were implanted, the stent frame would cover the bypass inflow, making access difficult in case of emergency and the need for surgery, including an unexpected ascending aortic procedure in case of IE recurrence. As a limitation of this case, despite no recurrence of infection 1 year after the surgery, the route of infection in the previous three PVEs has not been identified, and there is a possibility of recurrent PVEs, so vigilant follow-up is required. In addition, the Freestyle valve has the potential risk of causing structural abnormalities such as pseudoaneurysms due to inadequate tissue fixation with an immune response [25], or coronary artery stenosis from pseudo-intimal membranes [26]. Dagnegard et al. [27] reported conducting four-dimensional CT on patients post-Freestyle valve implantation, frequently identifying such structural issues. Although the patient’s preoperative CT did not reveal a pseudoaneurysm or RCA stenosis, repeated PVEs and THV implantation may lead to such complications in the future. Routine CT imaging is extremely important, because these problems are difficult to discern using TTE.

## Conclusions

We documented a case in which a patient survived three PVE episodes after the initial Bentall surgery utilizing a stentless valve and eventually developed SVD. A thorough examination of the patient’s history and TEE findings indicated a healed PVE. Considering the patient’s background, TAVR was performed. TAVR may benefit patients with limited IE lesions and a history of high reoperation risk.

### Supplementary Information

**Additional file 1: Video 1**. TEE findings on admission. TEE: transesophageal echocardiology.

**Additional file 2: Video 2**. TEE findings at the time of diagnosis of each PVE. TEE: transesophageal echocardiology, PVE: prosthetic valve endocarditis.

**Additional file 3: Video 3**. Transcatheter aortic valve in the full root stentless valve.

## Data Availability

The data sets generated and/or analyzed in the current study are available from the corresponding author upon reasonable request.

## Abbreviations

AI: Aortic insufficiency
AV: Aortic valve
BNP: B-type natriuretic peptide
CT: Computed tomography
IE: Infective endocarditis
MRSA: Methicillin-resistant *Staphylococcus aureus*
PVE: Prosthetic valve endocarditis
PVL: Paravalvular leak
RCA: Right coronary artery
SVD: Structural valve deterioration
SVG–LAD: Saphenous vein graft to left anterior descending artery
TAV-in-SAV: Transcatheter aortic valve implantation in a surgical aortic valve
TAVR: Transcatheter valve replacement
THV: Transcatheter heart valve
TEE: Transesophageal echocardiography
TTE: Transthoracic echocardiography
